# Strong Generative Capacity and the Empirical Base of Linguistic Theory

**DOI:** 10.3389/fpsyg.2017.01617

**Published:** 2017-09-21

**Authors:** Dennis Ott

**Affiliations:** Department of Linguistics, University of Ottawa Ottawa, ON, Canada

**Keywords:** generative grammar, grammaticality, acceptability, evidence, methodology

## Abstract

This *Perspective* traces the evolution of certain central notions in the theory of Generative Grammar (GG). The founding documents of the field suggested a relation between the grammar, construed as recursively enumerating an infinite set of sentences, and the idealized native speaker that was essentially equivalent to the relation between a formal language (a set of well-formed formulas) and an automaton that recognizes strings as belonging to the language or not. But this early view was later abandoned, when the focus of the field shifted to the grammar's strong generative capacity as recursive generation of hierarchically structured objects as opposed to strings. The grammar is now no longer seen as specifying a set of well-formed expressions and in fact necessarily constructs expressions of any degree of intuitive “acceptability.” The field of GG, however, has not sufficiently acknowledged the significance of this shift in perspective, as evidenced by the fact that (informal and experimentally-controlled) observations about string acceptability continue to be treated as *bona fide* data and generalizations for the theory of GG. The focus on strong generative capacity, it is argued, requires a new discussion of what constitutes valid empirical evidence for GG beyond observations pertaining to weak generation.

## Introduction

There exists a contradiction between the near-universal acceptance of acceptability judgments as a source of data for Generative Grammar (GG) on the one hand and the theory's express focus on *strong generative capacity* on the other. While linguists agree on this focus, they nevertheless tend to uncritically assume that judgments of the acceptability of strings constitute data for GG. But this assumption is baseless, and a renewed discussion of GG's empirical basis is in order.

## Early idealizations: the speaker as an automaton

Chomsky (1955, *LSLT*) defined as the “primary concern” of syntactic theory “to determine the grammatical sentences of any given language […]” (57). Chomsky (1957, *SS*) elaborates:

“The fundamental aim in the linguistic analysis of a language *L* is to separate the grammatical sequences which are sentences of *L* from the ungrammatical sequences which are not sentences of *L* […]. The grammar of *L* will thus be a device that generates all of the grammatical sequences of *L* and none of the ungrammatical ones.” (*SS*, 13).

The set of sequences so determined “corresponds to the ‘intuitive sense of grammaticalness’ of the native speaker” (*LSLT*, 95); hence, “the sequences generated by the grammar as grammatical sentences must be acceptable, in some sense, to the native speaker […]” (*LSLT*, 101). The adequacy of a grammar can be assessed by “[determining] whether or not the sequences that it generates are actually grammatical, i.e., acceptable to a native speaker” (*SS*, 13). Consequently, “the linguist's task [is] that of producing […] a grammar [that generates] all and only the sentences of a language […]” (*SS*, 85).

On this *Early View* (EV), the idealized native speaker is the human equivalent of an automaton in the theory of formal languages, which accepts (recognizes) or rejects a given string depending on whether or not it is part of the set of legal sequences. While the importance of hierarchical structures underlying the sequences was recognized to be of central importance, the formal systems used at the time—Post-style rewrite rules plus transformational rules—ultimately enumerated strings (see Lasnik, 2000).

*LSLT* and *SS* took grammaticality (or degrees thereof, as argued in *LSLT*: chapter 5; also Chomsky, 1965, p. 148ff.) to be accessible to intuition. That the matter is more complex was explicitly acknowledged shortly after by Chomsky (1965, p. 11), who cautions that “[t]he notion ‘acceptable’ is not to be confused with ‘grammatical”’: while the former “belongs to the study of performance,” the latter “belongs to the study of competence […].” This is the standard distinction between grammaticality and acceptability, often not drawn properly even in technical papers (cf. Newmeyer, 1983, p. 51). A true shift in perspective, however, took place later, when the notion of *sentence*, understood as *sequence in L*, was eliminated altogether from the theory.

## A shift in perspective

Later works of Chomsky's are explicit in rejecting the EV and its view of the idealized native speaker as a human automaton. Perhaps the first clear articulation of this shift appears in Chomsky (1980), where we find the assertion that “[a GG] does not in and of itself determine the class of what we might choose to call ‘grammatical sentences’ […],” an unremarkable conclusion “once we recognize that the fundamental concepts are *grammar* and *knowing a grammar*, and that *language* and *knowing a language* are derivative” (p. 126).

This dismissal of the view of a *language* as a set of sentences is a corollary of the shift of the focus of attention from sentences to *structures*:

“For each sentence, the grammar determines aspects of its phonetic form, its meaning and perhaps more. […] [It] is said to ‘weakly generate’ the sentences of the language and to ‘strongly generate’ the structural descriptions of these sentences” (Chomsky, 1980, p. 220).

The grammar *strongly* generates structural descriptions (SDs), not strings; the latter can at best be said to be generated in some *weak* sense, in that the “phonetic form” associated by the grammar with any SD has sequential properties (Chomsky, 1990). Importantly, the grammar is now no longer taken to generate objects of which the property “acceptability” or “well-formedness” could be predicated (i.e., strings/sequences/sentences).

Chomsky goes further in suggesting that the focus on strong generative capacity (SGC) in fact *requires* the generation of “deviant” expressions, as a matter of empirical fact:

“[A] GG will not generate the set of sentences that a speaker-hearer will regard as acceptable; indeed, it is virtually a criterion of adequacy that it should not, since so many different factors enter into such judgments” (Chomsky, 1980, p. 274 fn. 54).

Chomsky (1986, p. 24) adds that “[a] GG is not a set of statements about externalized objects constructed in some manner,” to which he refers as “E(external)-language,” as opposed to the I(nternal)-language that constructs SDs underlying these objects (see already Chomsky, 1959, 1963). This move replaces the EV of the grammar as determining a set of *sentences* with one of grammar as determining *form-meaning correlations*:

“[When a person knows a language], we do not mean that he or she knows an infinite set of sentences […]; rather, what we mean is that the person knows what makes sound and meaning relate to one another in a specific way […]” (Chomsky, 1986, p. 27).

Consequently, it is “meaningless to ask whether [some intuitively “deviant” expression] is, or is not, a member of the E-language weakly generated by *L*; and nothing would follow from a discovery (or stipulation) one way or another” (Chomsky, 1990, p. 145).

Chomsky (1986, p. 29f.) explains the motivation for the EV with the influence of formal-language theory on the then-nascent field of GG, an analogy now explicitly dismissed:

“In the literature of [GG], the term ‘language’ has regularly been used for E-language in the sense of a set of well-formed sentences […]. The misleading choice of terms was, in part [due to] the confluence of two intellectual traditions: traditional and structuralist grammar, and the study of formal systems. […] But the study of formal languages was misleading in this regard. When we study [a formal language], we may take it to be a ‘given’ […] infinite class of sentences in some given notation. Certain expressions in this notation are well-formed sentences, others are not. […] It is easy to see how one might take over from the study of formal languages the idea that the ‘language’ is somehow given as a set of sentences […], while the grammar is some characterization of this infinite set […]. The move is understandable, but misguided; […] the E-language is not ‘given’.”

Chomsky and Lasnik (1993, p. 508) reiterate this dismissal of the EV:

“[A] ‘formal language’ in the technical sense [is] a set of well-formed formulas […]. Call such a set an E-language […]. In the theory of formal languages, the E-language is defined by stipulation, hence is unproblematic. But it is a question of empirical fact whether […] I-language generates not only a set of [structures] but also a distinguished E-language […]. [T]he concept of E-language […] has no known status in the study of language […].”

The field of GG ostensibly followed Chomsky in shifting the focus from strings to SDs and their properties. What is customarily ignored, however, is that such a shift leaves notions such as “acceptability” or “well-formed sentence” with no immediate relevance to the theory of SGC.

## Beyond acceptability

Chomsky (1986, p. 98ff.) illustrates the practical effects of this shift in focus with a concrete example. While (1), where *who* is displaced from the gap position, receives a straightforward interpretation in terms of an operator-variable dependency, (2) cannot be interpreted in this way.

1. (I know) [who [John [kissed _]]]
  (“which person *x* is such that John kissed *x*”)
2. (I know) [who [John [kissed Mary]]]

In (2), the *wh*-operator has no variable to bind, and consequently cannot be assigned an interpretation. Importantly, we cannot simply “neglect” the fronted *wh*-phrase and interpret (2) as meaning *(I know) John kissed Mary*, a fact that Chomsky attributes to the principle of Full Interpretation—an interface condition, in current parlance. Does this mean that we want to block generation of (2), while allowing generation of (1)? Chomsky explicitly denies this, arguing that such a move would redundantly replicate the effect of Full Interpretation. Consequently, both SDs in (1) and (2) are grammatical (generated by the grammar); the “deviance” of (2) is due to an extraneous principle of interpretation. But the fact that the string deriving from (2) is “deviant” *per se* is of no immediate concern to the theory of grammar.

Analogously, to use the famous example introduced in *LSLT* (145), the goal of the theory is not to construct a grammar that generates a set of well-formed formulas including *Colorless green ideas sleep furiously* but excluding *Furiously sleep ideas green colorless*, but to explain why the SD assigned to the latter cannot be mapped onto an analogous interpretation. The naturalness of the typographical or acoustic object is of no immediate relevance to the theorist (cf. McCawley, 1982, p. 78f.). Similarly, island constraints are not generalizations over classes of sentences that are “unacceptable,” but describe the absence of otherwise expectable interpretations of expressions. The fact that *What does John like apples and?* is an intuitively “unacceptable” string is a mere observation; what does constitute a relevant explanandum for GG is solely the fact that it unexpectedly fails to mean “which *x* is such that John like apples and *x*?” (*pace* Preminger, in press).

On this *Revised View* (RV), the empirical success of GG depends on its ability to correctly model the speaker's knowledge of sound-meaning relations, not the intuitive acceptability of strings:

“Linguistic expressions may be ‘deviant’ along all sorts of incommensurable dimensions, and we have no notion of ‘well-formed sentence’ […]. Expressions have the interpretations assigned to them by the performance systems in which the language is embedded: period” (Chomsky, 1993, p. 27).

In later works, Chomsky entertains the idea that generation of SDs proceeds freely via the operation Merge, with constraints imposed only by external systems. For instance, Chomsky (2004, p. 111) argues that “theta-theoretic failures at the interface do not cause the derivation to crash; such structures yield ‘deviant’ interpretations of a great many kinds.” The relevant “theta-theoretic failures” are interface properties of SDs that are strongly generated, regardless of the deviance of derivative stimuli they may incur. More generally:

“Merge can apply freely, yielding expressions interpreted at the interface in many different kinds of ways. They are sometimes called ‘deviant,’ but that is only an informal notion. […] The only empirical requirement is that [the interfacing systems] assign the interpretations that the expression actually has, including many varieties of ‘deviance”’ (Chomsky, 2008, p. 144).

Chomsky (2016, p. 3f.) notes that “[f]ree application of rules can yield deviant expressions, but that is unproblematic, in fact required. Deviant expressions should be generated with their interpretations […],” as “[i]t would radically complicate the generative procedure if [Merge] were required to yield non-deviant structures,” “even assuming that the concept [of deviance, D.O.] can be defined in absolute terms, which has never been obvious” (fn. 8).

On this RV, there exists no notion of well-formedness that is given independently of whatever is strongly generated by the I-language. The grammar does not specify a set of legal strings but an infinity of SDs; the only empirical success criterion is that the SDs postulated by the theorist have the properties in interpretation and externalization they do.

## Quo vadis?

While the field ostensibly embraced the focus on SGC and SDs championed by Chomsky, the EV remains widely adopted in actual practice. Grammaticality and acceptability are standardly equated, and I-languages taken to determine sets of well-formed strings/sentences. The following quotes, randomly culled from popular textbooks, are representative:

“We say that an utterance is *grammatical* if native speakers judge it to be a possible sentence of their language” (O'Grady and Archibald, 2016, p. 139).

“The psychological experiment used to get to [the speaker's knowledge of language] is called the grammaticality-judgment task. The judgment task involves asking a native speaker to read a sentence, and judge whether it is well-formed (grammatical), marginally well-formed, or ill-formed (unacceptable or ungrammatical)” (Carnie, 2013, p. 14).

“[A] sequence of words is called a string. Putting a star at the start of a string is a claim that it isn't a grammatical sentence of the language in question” (Adger, 2003, p. 4).

“A […] reason for using grammaticality judgments [*sic*] is to obtain a form of information that scarcely exists within normal language use at all—namely, negative information, in the form of strings that are not part of the language” (Schütze, 1996, p. 2).

In a survey of empirical methods, Schütze (2011, p. 207) identifies the assumption “that our mental grammar distinguishes at least two kinds of strings: those that are possible sentences of our language and those that are not” as “Chomsky's view,” despite the fact that Chomsky has defended the opposite for at least 40 years.

As a result of this (unconscious?) adherence to the EV, acceptability judgments continue to take center stage in GG, and a good deal of the literature on experimental syntax has been devoted to refining their elicitation (Sprouse, 2013). Sprouse (2007, p. 123) notes that experimental methods have made it “almost trivial to detect subtle differences along a continuous spectrum of acceptability,” which he takes to raise the question of “whether the working assumption of the past 40 years should be abandoned”—this being the assumption “that grammatical knowledge is categorical—sentences are either grammatical or ungrammatical.” He explains that “the psychological claim underlying theories of categorical grammaticality is that ungrammatical sentences have no licit representation, [i.e.] cannot be constructed from the available mental representations.” There is no recognition of the fact that there exists no notion of “(un)grammatical sentence” on the RV, or any argument to the contrary.

The above remarks illustrate that the profound implications of the RV and its focus on generation of SDs remain insufficiently appreciated (cf. Fukui, 2015), and that the field's continuing obsession with string acceptability betrays the lasting impact of the EV. Technical work in GG remains strongly dominated by the assumption that syntactic computation ought to be virtually or entirely “crash-proof,” generating all and only those expressions that give rise to strings that are acceptable to the native speaker (*modulo* performance-related factors). This view is most explicitly espoused by Frampton and Gutmann (2002, p. 90), who maintain that “an optimal derivational system […] is a system that generates only objects that are well-formed and satisfy conditions imposed by the interface systems.” Note the use of the term “objects,” intended to ambiguously cover both sentences (the focus of the EV) and SDs and their semantic and phonological correlates (the focus of the RV).

This conceptually confused fixation on “crash-proofness” has given rise to a plethora of proposals that enrich the syntactic machinery in order to avoid “overgeneration” (e.g., by blocking certain extractions), ignoring the fact that this notion has no obvious relevance on the RV. A direct outgrowth of this ideology is the extensive reliance on highly stipulative features as licensors of structure-building (Chomsky, 2001, p. 6), leading to a “highly baroque syntax” (Reinhart, 2006, p. 5) employing “diacritic features that have no detectable properties other than their ability to trigger [syntactic operations]” (Richards, 2016, p. 1). Space precludes further discussion of the technical literature here; see Ott and Šimík (in progress).

The methodological problem posed by acceptability judgments, no matter how experimentally refined, is not their informal and inherently behavioral nature (Bever, 1970), but the fact that they do not constitute explananda for a theory of I-language (as opposed to E-language). The shift from the EV to the RV, traced above, demands a focus on speakers' knowledge of form-meaning correlations rather than string acceptability. Of course, in many cases “acceptability judgments” are in fact shorthand for judgments about such correlations—we can say that *He*_*i*_ *likes John*_*i*_ is “unacceptable,” or that it lacks the intended reading; we can say that (2) above is “deviant,” with an implicit understanding that we're referring to the absence of an interpretation analogous to (1). This innocent informal usage aside, however, the “(un)acceptable” status of sentences remains the de-facto empirical benchmark for theoretical proposals within GG, and informal observations about weak generative capacity, clad in technical terms, are standardly elevated to the status of generalizations to be accounted for (cf. the case of islands mentioned above). The field must overcome these limitations and move on to a theoretical characterization of possible SDs (e.g., in terms of the theory of Merge) and their interface properties (Chomsky et al., 2017). This will require the recognition that fears of “overgeneration” are unfounded, and more generally that GG's object of inquiry is much more abstract than the EV and its convenient idealizations suggested.

## Conclusion

The theory of GG has undergone significant conceptual shifts. Early work construed a GG as a finitary procedure that recursively enumerates all and only well-formed sentences of a language. Later work abandoned this conception entirely in favor of generation of discrete, hierarchically structured objects (I-language). Despite this shift, the field has retained a methodological obsession with the intuitive well-formedness of strings and associated notions such as “overgeneration” (E-language).

Chomsky (1965, p. 63) noted that “discussion of weak generative capacity marks only a very early and primitive stage of the study of [GG]. Questions of real linguistic interest arise only when [SGC] […] becomes the focus of discussion.” It is high time that this remark be taken seriously, which will necessitate a renewed discussion of the field's goals and the question of which observations can be translated into valid explananda for the theory, as opposed to mere translation of these observations into technical vocabulary. This will likely require the incorporation of various forms of evidence, from introspective to neurological, that can be hoped to tap the human “notion of structure,” in Jespersen's famous formulation.

## Author contributions

The author confirms being the sole contributor of this work and approved it for publication.

### Conflict of interest statement

The author declares that the research was conducted in the absence of any commercial or financial relationships that could be construed as a potential conflict of interest.
